# The protective effect of soybean protein‐derived peptides on apoptosis via the activation of PI3K‐AKT and inhibition on apoptosis pathway

**DOI:** 10.1002/fsn3.1776

**Published:** 2020-07-16

**Authors:** Guofu Yi, He Li, You Li, Fen Zhao, Zhiwei Ying, Menglan Liu, Jian Zhang, Xinqi Liu

**Affiliations:** ^1^ Beijing Advanced Innovation Center for Food Nutrition and Human Health Beijing Engineering and Technology Research Center of Food Additives Beijing Technology and Business University (BTBU) Beijing China

**Keywords:** apoptosis pathway, MG132, PI3K‐AKT pathway, soybean protein‐derived peptides

## Abstract

Soybean protein‐derived peptides (SBP) are a rich source of various bioactive peptides with multiple health benefits. However, the prospective effects of SBP on human cells are still unclear. Therefore, this article investigated the effects of small molecular weight SBP on MG132‐induced apoptosis in RAW264.7 cells. SBP inhibited MG132‐induced apoptosis of RAW264.7 cells in a dose‐dependent manner by flow cytometry. To further study its molecular mechanisms, Western blot analysis demonstrated that SBP could activate the PI3K‐AKT pathway by increasing the phosphorylation of PI3K and AKT and inhibiting apoptosis pathway by downregulating the expressions of pro‐apoptotic proteins of Bim, Bax, Fas, and Fasl and promoting the expressions of anti‐apoptotic proteins of Bcl‐xL and Bcl‐2. These results indicated the protective effect of SBP on MG132‐induced apoptosis in RAW264.7 cells.

## Graphical abstract

Soybean peptides display anti‐apoptosis effect in RAW264.7 cells through the activation of PI3K‐AKT pathway or indirect binding to Fas and FasL receptor.

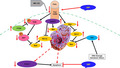

## 1 INTRODUCTION

Apoptosis is a process in which multiple genes are strictly controlled. These genes are conserved among species, such as the Bcl‐2 family, the caspase family, and the tumor suppressor gene P53. The Bcl‐2 protein family is mainly located in the mitochondria, endoplasmic reticulum, and continuous perinuclear membrane. Some members in this family, such as Bad, Bid, Bax, and Bim, promote apoptosis. These proteins can reside in the cytoplasm and receive the signal transduction after death, then translocated into mitochondria, promote cytochrome c release and apoptosis (Brenner & Mak, 2009; Razaghi, Heimann, Schaeffer, & Gibson, 2018; Whelan, Kaplinskiy, & Kitsis, 2010). After DNA damage, p53 can also be activated to induce Bax transcription (Seo, Lee, & Song, 2016). Some proteins prevent apoptosis, such as Bcl‐2, Bcl‐xL, which reside in the mitochondrial outer membrane, inhibit cytochrome c release and apoptosis (Hanahan & Weinberg, 2011). Caspase‐3 is a member in the family of cysteine proteases and a central regulator of apoptosis. Fas is activated by FasL, resulting in activation of caspase‐3. Upon activation of caspase‐3, the downstream effector cleaved caspase‐3 is hydrolyzed and activated for apoptosis (Theas, Rival, & Lustig, 2003). Fas receptor also plays a key role in caspase‐8 activation which is an important step in the initiation of the extrinsic pathway (Sobrido‐Camean & Barreiro‐Iglesias, 2018). After activation, caspase‐8 is released to the cytosol and initiates downstream apoptosis directly by activating caspase‐3 or indirectly through the mitochondrial pathway (Fritsch et al., 2019). However, cell survival also requires to actively control apoptosis by inhibiting the expression of pro‐apoptotic factors and promoting the expression of anti‐apoptotic factors (Altomare & Testa, 2005; Yap et al., 2011). The PI3K‐AKT pathway is an intracellular signaling pathway regulates many normal cellular processes including cell proliferation, survival, growth, motility, and longevity. Multiple survival factors activate the PI3K pathway, leading to activation of AKT, which plays an important role in cell survival signaling (Nitulescu et al., 2016). PTEN can negatively regulate the PI3K‐AKT pathway (Seo, Lee, Sung, et al., 2016). Activated AKT inhibits the pro‐apoptotic Bcl‐2 family members Bad and Bax (Luo, Budihardjo, Zou, Slaughter, & Wang, 1998; Palmer, 2003).

Abnormal apoptosis can affect the balance of cell growth and death, leading to organ dysfunction. The cell initiates an apoptotic program in the presence of environmental pollution (China‐Smog), radiation (Fukushima Daiichi disaster), genetic diseases, malnutrition, or other types of cell damage caused by severe stress cause premature cell apoptosis and damage to health. Inhibiting cell apoptosis can delay cell aging, which is beneficial to health and longevity (Hoda & Hoda, 2005). It has been reported that the active peptides of natural products can inhibit apoptosis. BH4‐domain peptide from Bcl‐xL exerted anti‐apoptotic activity in vivo (Sugioka et al., 2003). Selenoprotein hydrolysate (SPH) significantly reduced Pb^2+^‐induced caspase‐3 activation, reversed Pb^2+^‐induced Bax upregulation and cytochrome corelease, and downregulated intracellular Bcl‐2 expression (Fang et al., 2017). Nicolai Gronne Jorgensen et al have used a peptide derived from an anti‐apoptotic protein to perform a Phase I test of peptide vaccination on multiple myeloma with no adverse effects (Jørgensen et al., 2016). Liu et al. (2014) reported that Trp‐Asn‐Trp‐Ala‐Asp, a pentapeptide derived from egg white ovomucin pepsin hydrolysates, upregulated the level of anti‐apoptotic protein Bcl‐2 and downregulated the pro‐apoptotic protein level. Soybean protein‐derived peptides (SBP) are a rich source of various bioactive peptides and associated with many potential health benefits, including reducing the risk of obesity, high cholesterol levels, cardiovascular disease, insulin‐resistance/type II diabetes, certain types of cancers, and immune disorders (Kim et al., 2013, 2018; Li, Luo, & Zhang, 2014; Rayaprolu, Hettiarachchy, Horax, Kumar‐Phillips, et al., 2017). SBP like lunasin and soymorphins possess more than one of these properties and play a role in the prevention of multiple chronic diseases (Chatterjee, Gleddie, & Xiao, 2018). SBP also induce stem cell proliferation by mediating signal transduction, including activation of ERK and TGF‐β1 pathways (Lee, Roh, Kim, Lee, & Park, 2012). The external interventions, including environmental pollution, food safety, drug side effects, etc., can lead to accelerated cell death, resulting in various diseases that cause harm to human body. Proteins extracted from soy can be hydrolyzed by proteases to produce SBP with biological functions. SBP have been identified in different experimental systems to have biological properties (Rayaprolu, Hettiarachchy, Horax, Phillips, et al., 2017).

In this study, SBP were obtained by enzymatic reaction of soybean protein isolate (SPI). The obtained SBP having a molecular weight of 189–1,000 Da was then evaluated for its preventive effect on MG‐132‐induced apoptosis of RAW264.7 cells. Which cell signaling pathway from SBP used to inhibit RAW264.7 cell apoptosis or damage were determined by Western Blot. It provides a theoretical basis for SBP to inhibit premature cell apoptosis caused by external factors.

## 2 MATERIALS AND METHODS

### 2.1 Materials

**FIGURE 1:**
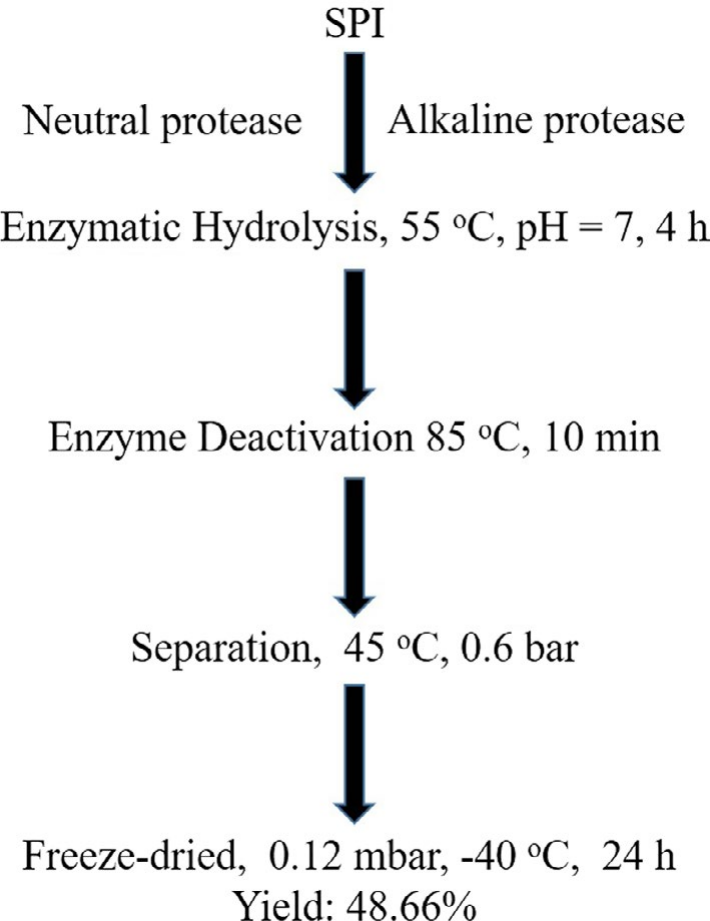
Preparation of SBP from SPI

The soybean protein isolate (SPI) used in this study were provided by Nutrily Biotechnology, Ltd. (Patent No.: CN107674900A) and SBP were prepared according to the method described (Liu & Scotland, 2017; Zhao et al., 2018). As given in Figure 1. The primary antibodies against Bax (2772S), Bcl‐2 (3498S), Caspase‐3 (9662S), cleaved caspase‐3 (9664S), AMPKα (5832S), Phospho‐AMPKα (2535S), PTEN (9188S), p53 (2524S), PI3K (4249S), P‐PI3K (4228S) AKT (4685S), p‐AKT (9611S), Bad (9268S), GAPDH, were purchased from Cell Signaling Technology. Bim antibody (orb87294), Caspase‐8 Antibody (orb10664) and cleaved‐Caspase8 Antibody (orb159341) were obtained from Biorbyt. Both HRP‐conjugated Goat anti‐mouse or rabbit secondary antibodies were purchased from Jackson Labs Technologies.

### 2.2 Determination of SBP molecular weight distribution

The measurement was performed on the LC20A by gel filtration chromatography. The column used was a TSK‐GEL G2000SWXL column (5 μm, 7.8 mm × 300 mm). The mass concentration of the prepared SBP is 1 mg/ml, and the preparation concentration of all molecular weight standards is 1 mg/ml. Before injection, samples and standards (Gly‐Gly‐Gly, Gly‐Tyr‐Arg, bacitracin, and insulin) were filtered through a 0.22 μm filter. The injection volume was 20 µl, the mobile phase was *V*(water)/*V*(acetonitrile)/*V*(trifluoroacetic acid) = 80:20:0.1, the flow rate was 0.5 ml/min, and the detection wavelength was 220 nm.

### 2.3 Cell culture

RAW264.7 cells were maintained in Dulbecco's modified Eagle's medium (DMEM), supplemented with 10% fetal bovine serum (FBS) and 1% penicillin/streptomycin in a 5% CO_2_ incubator. Cells were digested with 0.25% trypsin solution for cell passage.

### 2.4 MG‐132 and SBP treatment modeling and cell viability tests

To establish cellular apoptosis model, RAW264.7 cells were seeded into 6‐well plate at a density of 4 × 10^5^ cells/ml. MG132 was dissolved in the medium with the concentration (0, 3, 5, 10, 20, 30, 40, 50 and 60 μM) for 4 hr. Then, cells were collected for viability detection. Cell viability was determined by MTT assay. To assess the effect of SBP on cellular apoptosis, RAW264.7 cells were pretreated with 10 ml of fresh DMEM containing different concentrations of SBP substrates (0.00, 0.06, 0.13, 0.25, 0.50, 1.00 and 2.00 mg/ml) followed by MG‐132 treatment. Then, cells were collected for viability detection. Cell viability was determined by MTT assay. Briefly, 4 × 10^5^ cells/ml were plated in 96‐well plates a day before SBP or MG132 treatment. After pretreatment by SBP (24 hr) or MG132 (4 hr), MTT solution (20 μl, 5 mg/ml) was added to each well to dissolve formazan crystals. Absorbance was measured at 450 nm by using a plate reader (Bio‐Rad Laboratories Ltd.).

### 2.5 Apoptosis detection of RAW264.7 cells by flow cytometry

RAW264.7 cells were placed in 6‐well plates at a density of 4 × 10^5^ cells/ml and treated with different concentrations of SBP substrates (0.13, 0.25, and 0.50 mg/ml). After incubation of 24 hr, cells were treated with MG‐132 at the concentration of 1 mg/ml for 4 hr. The cells were harvested by trypsinization, washed twice with precooled PBS, and centrifuged at 1,000 *g* for 5 min to collect cell pellets. They were resuspended by adding 150 μl 2 × binding buffer. Then 5 μl of annexin V‐FITC was added and incubated with the cells at room temperature for 20 min. Add 5 μl PI to the cells for 5‐min incubation, and then 100 μl 2 × sample buffer before transferring to the machine. Fluorescence intensity reflecting apoptosis rate was measured using a CytoFLEX FCM (Beckman).

### 2.6 Western Blot analysis

RAW 264.7 cells were collected and lysed by ice‐cold RIPA and incubated on ice for 30 min. Cell proteins were extracted, and total protein concentration was measured by BCA method and adjusted to a protein concentration of 2 μg/μl. Each protein sample was subjected to SDS‐PAGE electrophoresis in a volume of 5 μl and transferred to the membrane. After that, it was blocked with 5% BSA for 1–2 hr at room temperature, and then washed with TBST for three times, each time for 5 min. Then, each primary antibody was added, and incubation of the primary antibody was at 4°C overnight. The incubation of HRP‐conjugated secondary antibody was for 1.5 hr at room temperature. At last, the ECL substrate was applied to the blot for image capture. GAPDH was used as a loading control.

### 2.7 Data analysis

All experiments were repeated three times and the results were expressed as mean and standard deviation (*SD*). One‐way analysis of variance (ANOVA) was performed on all data using SPSS 16 software. A level of *p* value < .05 was considered statistically significant.

## 3 RESULTS

### 3.1 Characteristics of SBP

The Shimadzu LC20A instrument was operated by gel filtration chromatography to determine the molecular weight distribution of SBP. As shown in Table 1, the ratio of molecular weight 189–1,000 Da was observed to be 84.01%, the average molecular weight was about 723.12 Da, and consisted of about 3–6 amino acids.

**TABLE 1:** Molecular weight distribution of SBP

Molecular mass range/Da	Integral area (%)	Proportion (%)	Average relative molecular mass/Da
>3,000	0.52	15.41	723.12
3,000–1,500	1.19		
1,500–1,000	13.22		
1,000–500	61.23	84.01	
500–189	23.25		
<189	0.59	0.58	

### 3.2 Effect of MG‐132 and SBP on cell viability

**FIGURE 2:**
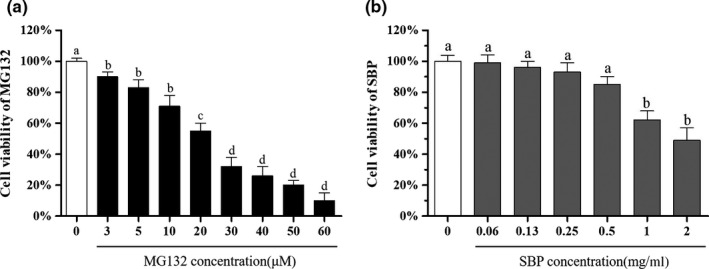
Effects of MG‐132 on the viability of RAW264.7 cells (a), and the effects of SBP on the viability of RAW264.7 cells (b). All results were expressed as the means ± *SD* (*n* = 3). There was no significant difference in the results of the same letters (*p* > .05)

Cell viability is an important indicator of cell survival or death after exposure to toxic substances or stimulation. This process involves multiple pathways and mechanisms, which are reflected in alterations in certain gene and protein expression (Ji, Zhang, Shi, & Hou, 2011; Pan et al., 2014). As shown in Figure 2a, as the concentration of MG‐132 increased, cell viability decreased in a concentration‐dependent manner, and the inhibitory effect of MG‐132 on cell proliferation was gradually enhanced. When the concentration of MG‐132 was higher than 20 μM, the cell viability was significantly decreased (*p* < .05). When the cells were treated with a concentration of 20 μM MG‐132 for 4 hr, the cell survival rate was 58%. Therefore, 20 μM was selected as the concentration of MG‐132 treatment.

The MTT assay was performed to rule out the potential cytotoxic effects caused by the SBP. As shown in Figure 2b, the cell viability decreased in a concentration‐dependent manner with the increase of sample concentration. Cell viability decreased significantly (*p* < .05) when the SBP concentrations were 1.00 and 2.00 mg/ml, indicating severe RAW264.7 cell damage. Therefore, the appropriate concentration of the SBP was 0.50 mg/ml. In addition, two experimental samples were established using concentrations of 0.25 and 0.13 mg/ml to further investigate the protective effects of SBP on cells.

### 3.3 Effect of SBP on cellular apoptosis rate

**FIGURE 3:**
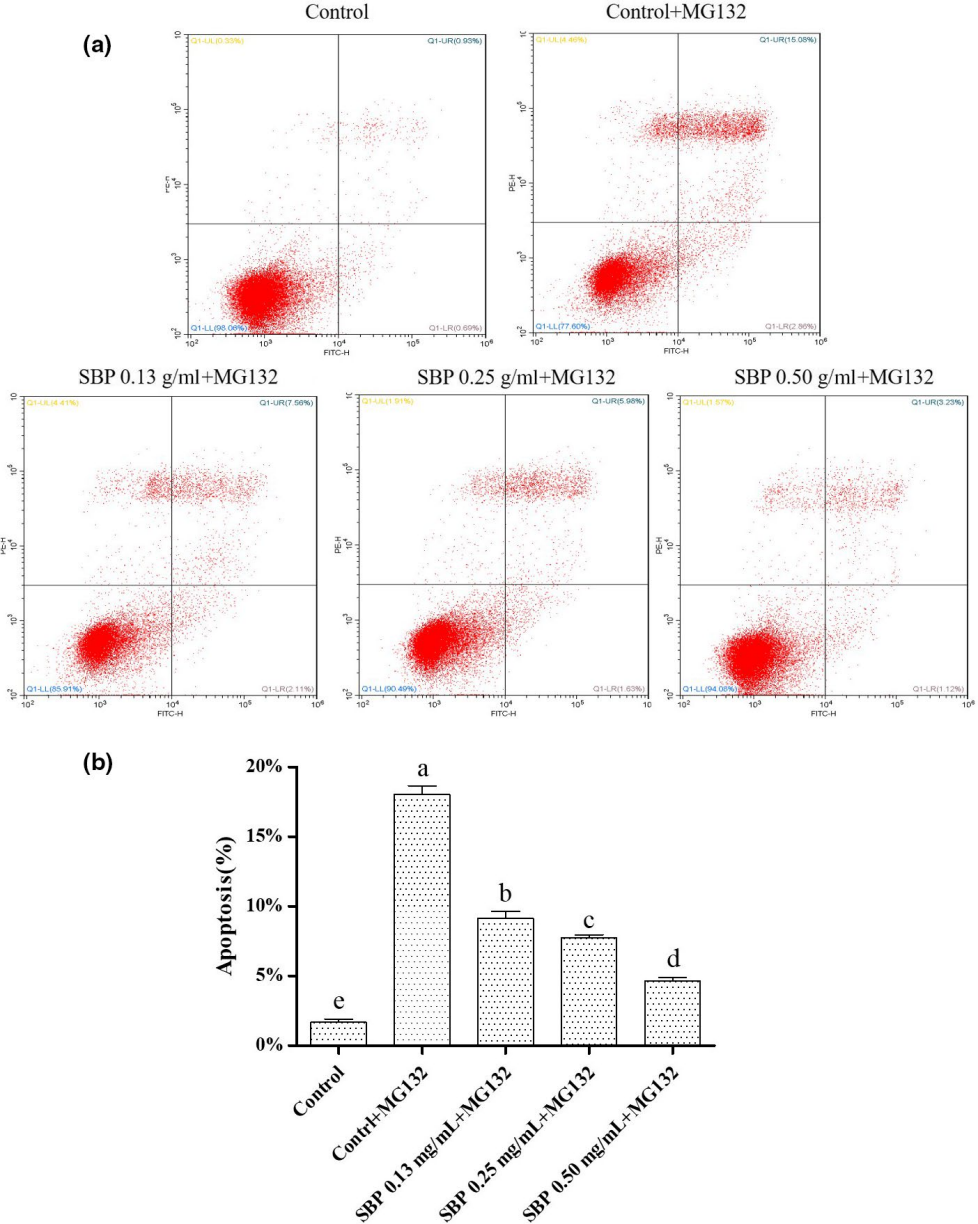
Apoptosis was detected by annexin V‐FITC staining using by flow cytometry (a). The histogram summarizes the characteristics of apoptosis changes (b). All results were expressed as the means ± *SD* (*n* = 3). There was no significant difference in the results of the same letters (*p* > .05)

The effect of SBP on RAW264.7 cell apoptosis treated with MG‐132 at different concentrations (0.13, 0.25, and 0.5 mg/ml) for 24 hr were evaluated. Apoptosis data obtained by flow cytometry, as shown in Figure 3a. The apoptosis rate of the control group was 1.67 ± 0.13%, the control + MG‐132 group was 18.04 ± 0.63%, the SBP 0.13 mg/ml + MG‐132 group was 9.15 ± 0.48%, the SBP 0.25 mg/ml + MG‐132 was 7.71 ± 0.14%, and the SBP 0.50 mg/ml + MG‐132 group was 4.62 ± 0.24%. The results as shown in Figure 3b, the apoptosis rate of the control + MG‐132 group was almost ten times higher than that in the control group. The apoptosis rate of control + MG‐132 was 1.97, 2.34, 3.92‐fold comparing to that groups under the intervention of three different SPB concentrations (0.13, 0.25 and 0.50 mg/ml). The results indicated that SBP treatment reduced the proportion of apoptotic cells in a dose‐dependent manner.

### 3.4 Effects of SBP on PI3K‐AKT pathway

**FIGURE 4:**
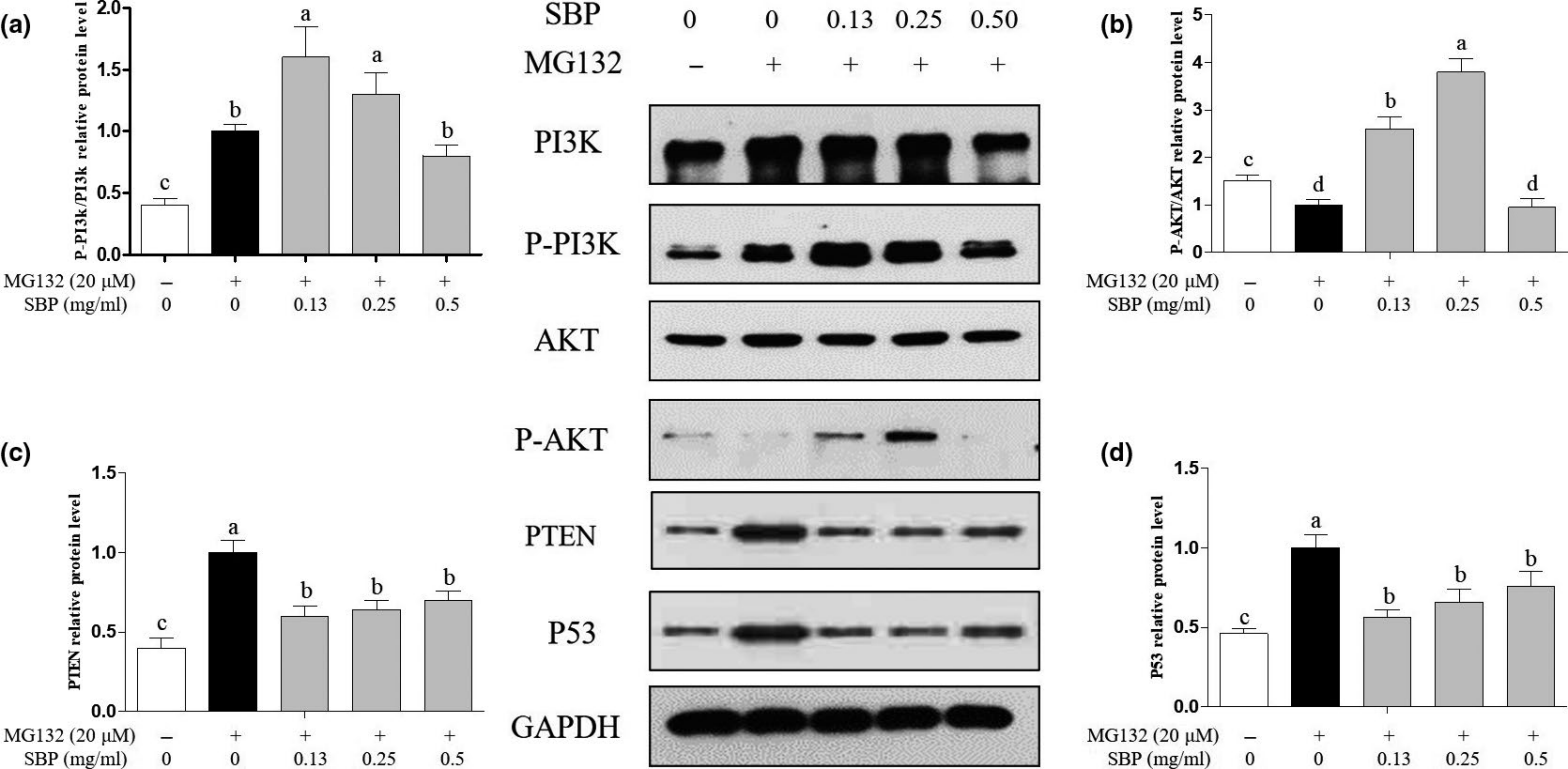
Detection of expression of PI3K‐AKT pathway related proteins in RAW264.7 cells induced by MG‐132 when treated with different concentrations of SBP. GAPDH was used as the reference gene

To understand the molecular mechanism of SBP on MG132‐induced apoptosis, we focused on the PI3K‐AKT pathway first. As shown in Figure 4a, compared with the blank group, MG‐132 significantly (*p* < .05) upregulated the expression of P‐PI3K/PI3K protein in the positive control group. Compared with the positive control group, 0.13 and 0.25 mg/ml SBP can significantly (*p* < .05) increase PI3K/P‐PI3K protein expression, while 0.50 mg/ml SBP had no effect on PI3K and P‐PI3K protein expression difference. As shown in Figure 4b, in the detection of P‐AKT/AKT, compared with the blank group, the expression of P‐AKT/AKT in the positive control group was significantly reduced under the treatment of MG‐132. Compared with the positive control group, 0.13 and 0.25 mg/ml SBP can significantly (*p* < .05) increase P‐AKT/AKT protein expression, while 0.5 mg/ml SBP had no significant difference. As shown in Figure 4c, compared with the blank group, the positive control group under the treatment of MG‐132, PTEN expression was significantly increased. Compared with the positive control group, SBP (0.13, 0.25, 0.5 mg/ml) can significantly (*p* < .05) reduce PTEN protein expression. As shown in Figure 4d, in the detection of P53 protein change, compared with the blank group, the positive control group under the treatment of MG‐132, P53 protein expression was significantly increased. Compared with the positive control group, SBP (0.13, 0.25, 0.5 mg/ml) can significantly (*p* < .05) reduce P53 protein expression.

### 3.5 Effects of SBP on exogenous apoptosis pathway

**FIGURE 5:**
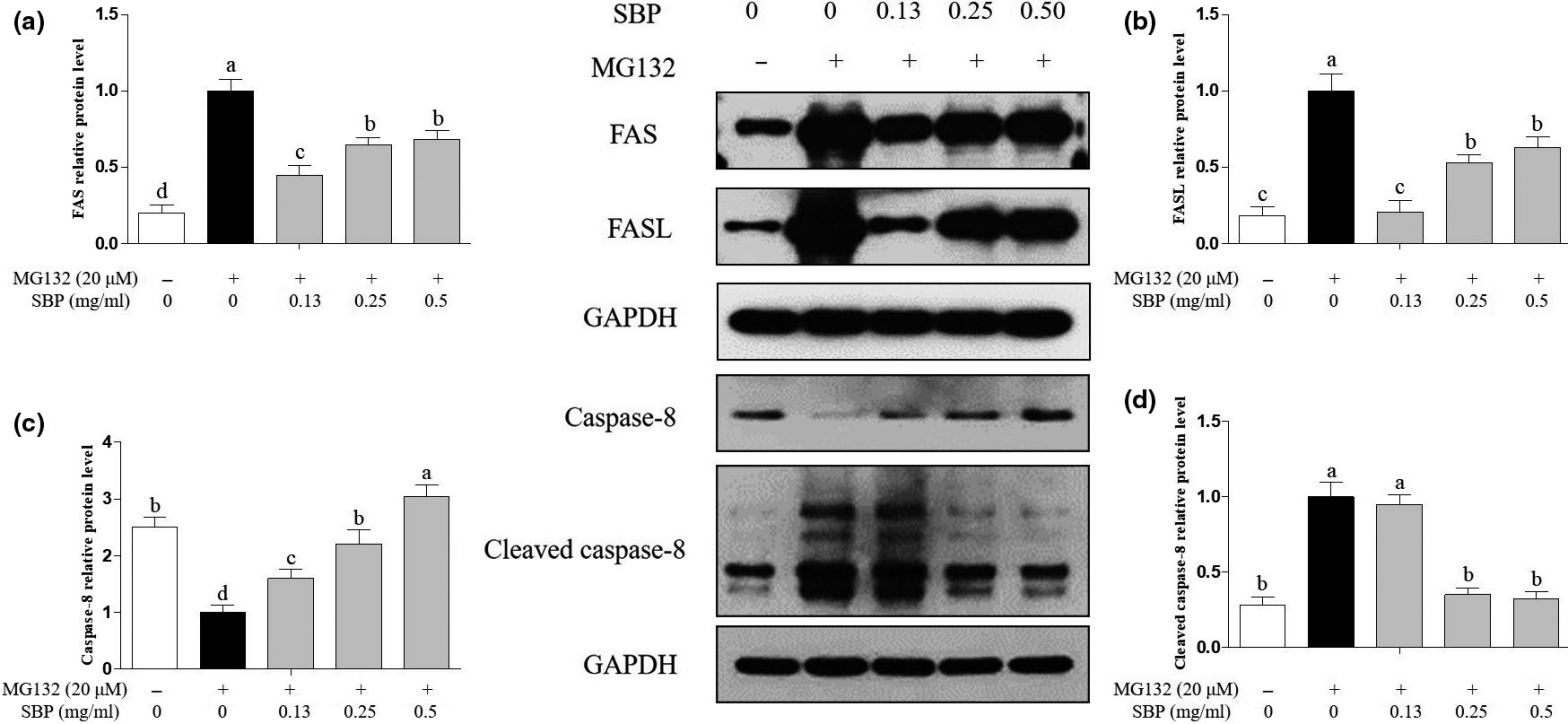
Expression of exogenous apoptosis related proteins were detected by WB.in RAW264.7 cells treated with MG‐132 and different concentrations of SBP. GAPDH was used as the reference gene

Next, the influence of SBP and MG132 on exogenous apoptosis was investigated by detection the expression levels of related proteins. As shown in Figure 5a, the expression of Fas protein increased by 3.4–3.7 times comparing to MG‐132 (*p* < .05), and decreased 1.2–2.4 times under the treatment of SBP (0.13, 0.25 and 0.50 mg/ml). As shown in Figure 5b, the expression of FasL protein was significantly increased 6.5–6.8 times by MG‐132 (*p* < .05), but decreased 1.2–4.8 times by SBP (0.13, 0.25 and 0.50 mg/ml) treatment comparing to MG‐132. For the downstream gene detection, as shown in Figure 5c,d, the expression of Caspase‐8 was downregulated by MG‐132 treatment for 2.3 times, which also indicated by the increase of Cleaved caspase‐8. The treatment of SBP (0.13, 0.25 and 0.50 mg/ml) displayed conspicuous inhibition on the expression of Fas and FasL by 0.6–1.2 time respectively, and SBP (0.13, 0.25 and 0.50 mg/ml) further influenced the cleavage of Caspase‐8 by 0.2–2.2 times. Because the soybean peptides used in our study were a mixture, the multiple components may regulate different target genes in different manners in this pathway, resulting in the differences on dose‐dependent manner in target gene expression. These WB results indicated the inhibition of SBP on the exogenous apoptosis pathway in RAW264.7 cells.

### 3.6 Effects of SBP on intrinsic apoptosis pathway

**FIGURE 6:**
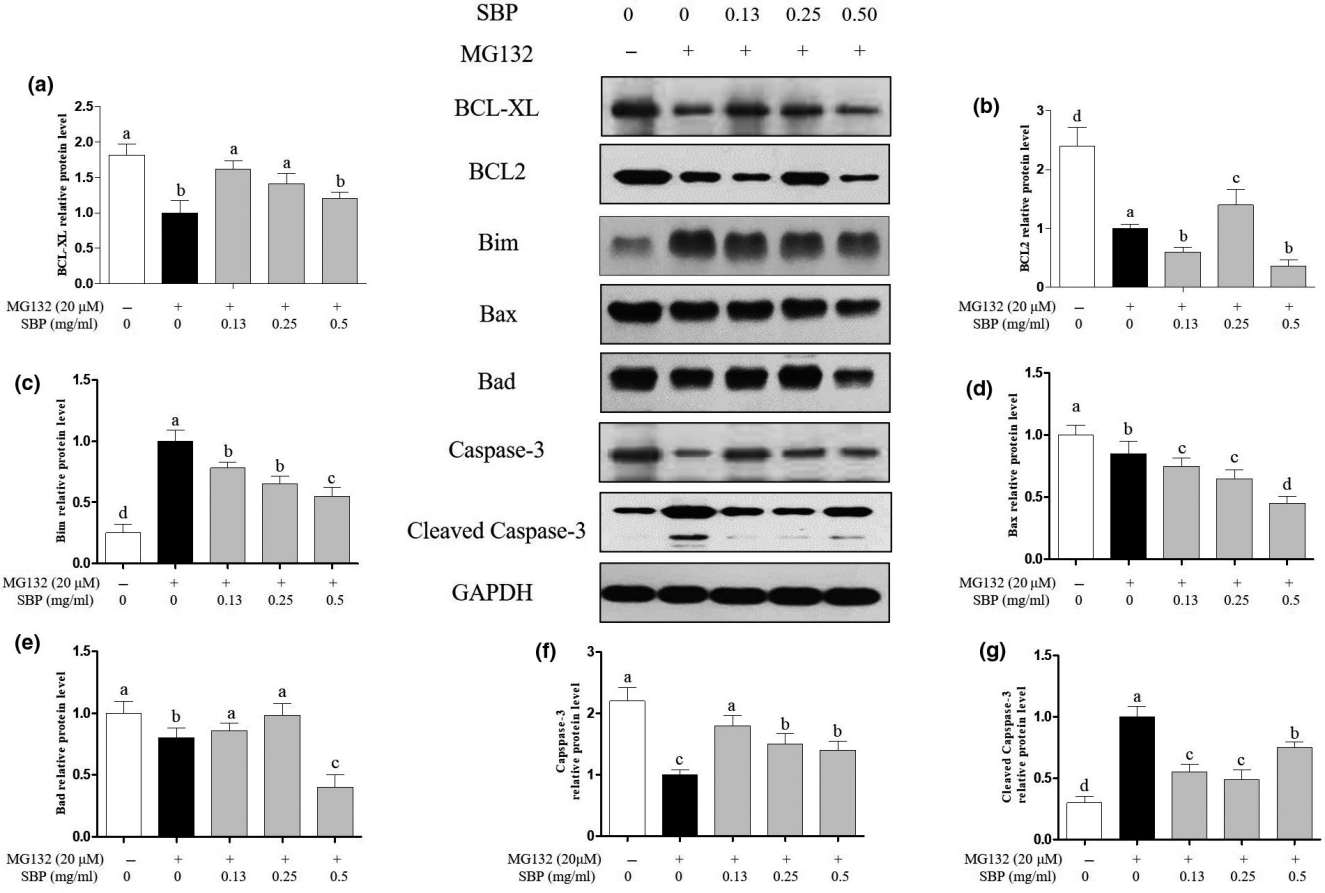
Expression of intrinsic apoptosis related proteins were detected by WB.in RAW264.7 cells treated with MG‐132 and different concentrations of SBP. GAPDH was used as the reference gene

To fully understand the apoptosis pathway, protein levels of the intrinsic apoptosis pathway were also detected in‐depth knowledge finally. The treatment of MG‐132 increased the expression of pro‐apoptosis proteins and inhibited the expression of anti‐apoptosis proteins. However, compared with MG132, the SBP inhibited the expression of pro‐apoptosis proteins and promoted the expression of anti‐apoptosis proteins. It seems that SBP had a strong inhibitory effect when they induced apoptosis in the external environment. As shown in Figure 6, the augment of cleaved Caspase‐3 manifested the activation of apoptosis pathway upon MG‐132 treatment along with the increase of pro‐apoptotic protein and decreased of inhibitor of apoptosis protein compared with the control group. Then, the pro‐apoptotic protein (Bim, Bax) was significantly (*p* < .05) decreased by 0.2–0.5 times respectively upon SBP addition in a dose‐dependent manner compared with group under the treatment of MG‐132, and the anti‐apoptotic protein (Bcl‐xL and Bcl‐2) increased by 1.3–3 times under the treatment of SBP (0.50 mg/ml). These results demonstrated significant protective effect of our SBP on the intrinsic apoptosis pathway.

## 4 DISCUSSION

To explore the molecular mechanism of SBP on MG‐132‐induced apoptosis, we investigated the influence of SBP on the PI3K‐AKT and apoptosis pathways in cells, and further studied expression levels of important proteins in these pathways. Some cytotoxic drugs, like cisplatin, gemcitabine, can trigger apoptosis by inducing G2/M arrest and p53 expression or preventing Myc protein expression (Hung et al., 2010). In our study, MG‐132 induced the apoptosis by regulating Fas‐mediated and mitochondria‐mediated protein expression. Based on the results of WB, the function of MG‐132 may bind to ligands on cellular surface and transit into cytoplasm inducing mitochondria membrane potential change which further triggers apoptosis. The results of FACS also confirm that both the early and later stages of apoptosis were induced by MG‐132. The extrinsic pathway directly activates the caspase cascade through the “death receptor” on cell surface. The most common death ligand is Fas ligand (Tannkulu‐Kucuk et al., 2019). Subsequently, apoptosis is initiated by cleavage of other downstream caspase, such as caspase‐3 (Iyer et al., 2015). The treatment of MG‐132 induced the expression of Fas and FasL, which indicated that activation of extrinsic apoptosis pathway. By down‐regulating the expression of Fas and FasL, SBP can protect cells from exogenous apoptosis, indicating that the active components in SBP can inhibit the expression of Fas and FasL (Liu, Hou, Zhang, & He, 2019). The effect of SBP on the mitochondrial apoptosis pathway is mediated by alterations of protein expressions located in mitochondria, which further destroy the permeability of mitochondria. The cytoplasmic release of cytochrome c activates caspase‐3 by forming an apoptotic body complex.

The protective effect of multiple SBP has been reported in detail. Kim et al. purified the hydrophobic SBP X‐MLPSYSPY from defatted SBP that arrested the cell cycle progression at G2/M phase in murine lymphoma cells (Kodaka et al., 2019). The low‐molecular‐weight protein Bowman–Birk protease inhibitor (BBI) can exert anti‐cancer activity involving apoptosis through reactive‐oxygen‐species‐induced mitochondrial damage after proteasomal inhibition and anti‐angiogenesis (Li, Dong, et al., 2014). Lunasin is another chemo preventive SBP that is closely associated with the BBI. Lunasin binds to nonacetylated H3 and H4 histones to prevent their acetylation, exerting anti‐carcinogenic activity (Davis & Inaba, 2016). In human breast cancer cell line MCF‐7, lunasin induced apoptosis in MCF‐7 cells by upregulation of tumor suppressor PTEN. Whereas, lunasin is also able to inactivate the tumor suppressor proteins, Rb, p53, and p32, and compete with the histone acetyltransferases to bind to the core deacetylated histones H3 and H4, and stop the transcription, leading to arrest of the G1/S phase and apoptosis (Jiang et al., 2016). Based on these reports, the influence of SBP has multiple levels of regulation effects on apoptosis.

Upon the SBP treatments, the protein alterations of Bad, Bim, BCL‐XL, Fas, and FasL were significantly dose‐dependent, while the alterations of other proteins were not dose dependence. The effect of SBP on apoptosis may also be an indirect influence of AKT pathway. AKT, accompanied with PI3K to block cell apoptosis by inhibiting pre‐apoptosis Bcl‐2 family members (Radogna & Diederich, 2018). Similar to Zhang's result, glucagon‐like peptide‐1 (GLP‐1) protects cardiomyocytes from AOPP‐induced apoptosis, mainly by increasing the expression of PI3K and AKT, and inhibiting the protein expression of Bad (Zhang et al., 2016). Generally, the activation of PI3K‐AKT pathway needs activator and receptor direct binding. This signaling leads to the activation of intrinsic tyrosine kinase, which further recruits and activates PI3K and downstream AKT signaling (De Luca, Maiello, D'Alessio, Pergameno, & Normanno, 2012). Many cytokines, such as IGF1, EGF, IGF2 and small SBP have the functions on PI3K‐AKT pathway activation by binding to different receptors in cellular surface. The SBP may have the similar structure or components of PI3K‐AKT pathway activator, which binds to IGFR or other receptor to further activate the pathway (Lin et al., 2017). On the other hand, short SBP of <20 amino acids can use endocytosis and translocation to internalize into cells, however, the internalization mechanism of these SBP in cells still remains largely controversial (Barone & Zimmer, 2016; Jiao et al., 2009). So, we also propose the hypothesis that SBP may utilize endocytosis or other internalization mechanism to permeate into RAW264.7 cell, which further bind to adaptor protein mTORC1 of PI3K‐AKT pathway, or apoptosis pathway regulator proteins to perform the protective effect on apoptosis induced by MG‐132.

Using different approaches, many soybean bioactive peptides have a variety of physiological functions. However, the exact components of SBP which possess the biological function were not isolated in our current study. It is a bit one‐sided to separate the mixed peptides to study their activity, which cannot fully represent peptides as functional foods. The efficacy of peptide mixtures is higher than that of a single peptide, nutrition is more comprehensive, and amino acids are more balanced. Especially as a functional food, it has a wide range of applications in assisting certain medical diseases. Among them, unhealthy diet nutrition is related to the development and deterioration of many disease states, thus explaining the important role of diet in nutrition balance (Kaminski et al., 2019). Therefore, this study may help to better understand the role of bioactive peptides in preventing injury. However, more studies are required to further identify their target organs and elucidate their biological mechanisms of action in order to be potentially used as functional food or even therapeutics for prevention or treatment of chronic diseases (Li‐Chan, 2015).

The present study evaluated anti‐apoptosis activity induced by MG‐132 of SBP on RAW264.7 cell. The supplement of SBP did not show cytotoxic activity, while MG‐132 stimulated strong apoptosis effect on RAW264.7 cell. SBP mediated anti‐apoptosis effects through the activation of PI3K‐AKT pathway, whereas inhibited the exogenous apoptosis pathways by down‐regulating Fas and FasL expressions. Furthermore, SBP also reduced the mitochondria‐mediated apoptosis pathway through regulating Bax and Bcl‐2 expressions. Results of this study suggested SBP has a novel anti‐apoptosis effect, which may broaden its application as functional food ingredients.

## 5 CONCLUSIONS

Soybean is a promising source of peptides with a wide range of biological activities. In the present study, soybean protein was hydrolyzed by mix proteases to produce SBP which display anti‐apoptosis effect in RAW264.7 cells through the activation of PI3K‐AKT pathway or indirect binding to Fas and FasL receptor. Further studies are needed for better understanding of the active ingredients of SBP, and their absorption, metabolism, and target tissues in animal models, as well as clarification of their mechanism of actions in other cellular signaling pathways.

## CONFLICT OF INTEREST

The authors declare no conflict of interest.

## ETHICAL APPROVAL

This article does not contain any studies with human participants or animals performed by any of the authors. Informed consent was obtained from all individual participants included in the study.
